# Supplementary material for: Public opinion about the UK government during COVID-19 and implications for public health: A topic modeling analysis of open-ended survey response data
Source: PLoS One. 2022 Apr 14;17(4):e0264134. doi: 10.1371/journal.pone.0264134 (PMC9009625; doi:10.1371/journal.pone.0264134)
Supplement: S1 Table — (DOCX) [file pone.0264134.s001.docx]

| Question |
| --- |
| *Q1. Is there anything you would like to tell us about the changes that have been brought about by the Covid-19 pandemic and the impact these have had on your mental health or wellbeing? |
| *Q2. What is bothering you the most about the pandemic? What aspects of it have you been finding most difficult? |
| *Q3. Has the pandemic had any negative impacts on your mental health and wellbeing? If so could you tell us about these? |
| *Q4. Has the pandemic had any positive impacts on your mental health and wellbeing? If so could you tell us about these? |
| Q5. How have your circumstances (e.g. work, housing, local area, finances, social networks, family life, responsibilities etc ) contributed to your experiences (positive, negative or both) of the pandemic? |
| Q6. How have your personal attributes (e.g. age, gender, ethnicity, sexuality, health conditions etc) contributed to your experiences (positive, negative or both) of the pandemic? |
| Q7. What have been your methods for coping during the pandemic so far and which have been the most or least helpful? |
| *Q8. Since the Covid-19 pandemic began, how have you been feeling about the future? What are you hopeful or concerned about? |
| * Question used in this analysis |
