# Supplementary material for: Public opinion about the UK government during COVID-19 and implications for public health: A topic modeling analysis of open-ended survey response data
Source: PLoS One. 2022 Apr 14;17(4):e0264134. doi: 10.1371/journal.pone.0264134 (PMC9009625; doi:10.1371/journal.pone.0264134)
Supplement: S2 Table — Includes spelling mistakes. (DOCX) [file pone.0264134.s002.docx]

| Keywords |
| --- |
| government (4902), government's (405), trace (396), govt (372), political (344), governments (300), track (290), politicians (248), cummings (183), scientists (144), scientific (143), politics (142), power (142), boris (128), westminster (123), dominic (103), gov (99), johnson (93), tax (86), authorities (83), taxes (83), tory (80), election (75), authority (63), austerity (62), ministers (50), democracy (49), advised (48), pm (48), minister (43), politically (42), sage (40), conservative (38), tracing (37), advisors (34), labour (33), powers (33), goverment (30), elected (29), cabinet (28), vote (28), voted (26), governmental (25), advise (24), parliament (24), elections (23), governed (23), gov't (19), scientist (19), dido (17), govt's (16), governance (15), elective (14), populist (14), democratic (13), politicised (13), advisers (12), conservatives (12), govern (12), populism (12), sturgeon (12), johnson's (11), taxpayers (11), governement (10), gvt (10), whitty (10), advising (9), authoritarian (9), authoritarianism (9), establishments (9), govenment (9), hancock (9), hmg (9), politician (9), tracked (9), parliamentary (8), pm's (8), politicising (8), taxation (8), voting (8), barnard (7), cummins (7), durham (7), establishment (7), ferguson (7), minister's (7), chancellor (6), goverments (6), politicisation (6), socialist (6), taxpayer (6), totalitarian (6), tracks (6), unelected (6), whitehall (6), adviser (5), chris (5), elect (5), electing (5), governing (5), libertarian (5), thatcher (5), advisor (4), advisory (4), autocracy (4), bojo (4), boris's (4), electorate (4), gove (4), governor (4), govts (4), hmrc (4), phe (4), referendum (4), scientifically (4), taxed (4), tracking (4), votes (4), borris (3), farage (3), governors (3), govnt (3), jenrick (3), politicise (3), politicking (3), populists (3), reelected (3), rightwing (3), rishi (3), sadiq (3), taxing (3), traced (3), voter (3), advises (2), antidemocratic (2), authoritative (2), bureaucracy (2), bureaucratic (2), bureaucrats (2), cabinets (2), democracies (2), democratically (2), electoral (2), givernment (2), gov.com (2), gov.uk (2), goverment's (2), governemtn (2), govmt (2), govn (2), gvmt (2), johnsons (2), johnston (2), libertarians (2), polical (2), politic (2), politicans (2), politicization (2), politicizing (2), socialism (2), sunak (2), sunak's (2), taxiing (2), toryscum (2), tracer (2), tracker (2), undemocratic (2), unscientific (2), westminister (2), antiestablishment (1), antiestablishmentarianists (1), atuhorities (1), auth9orities (1), authoritarians (1), authouritarian (1), autocratic (1), bj (1), burnham (1), burueacracy (1), cabinet's (1), chancellors (1), churchill (1), churchill's (1), conservaties (1), corbyn (1), cummingsgate (1), dcm (1), dcms (1), domini (1), dominick (1), domminic (1), domnic (1), doris's (1), e.g.bojo (1), etonians (1), farage's (1), from.government (1), givenment (1), gobernment (1), goernment (1), gorenment (1), gouvernement (1), govenmental (1), govenments (1), govenrnment (1), governmant (1), governmenet's (1), governmenst (1), government.think (1), governmentresponses (1), governments.this (1), governmentt (1), governmet (1), governmrnt's (1), goverrnment (1), govertment (1), govt.messages (1), gvnt (1), gvts (1), jenryk (1), jonson's (1), lefites (1), leftie (1), liarjohnson (1), parliaments (1), pms (1), policitians (1), politians (1), politicallu (1), politicians.politicians (1), politicitians (1), politicos (1), polititians (1), popularism (1), populistm (1), primeminister (1), priti (1), publichealth (1), reelection (1), rishi's (1), rulers (1), rulership (1), rushi (1), sage's (1), starmer (1), sunack (1), taxpayer's (1), thatcher's (1), thatcherite (1), torygraph (1), totalitarianism (1), tracers (1), westminster's (1), westminsters (1), williamson (1) |
