# Supplementary material for: Public opinion about the UK government during COVID-19 and implications for public health: A topic modeling analysis of open-ended survey response data
Source: PLoS One. 2022 Apr 14;17(4):e0264134. doi: 10.1371/journal.pone.0264134 (PMC9009625; doi:10.1371/journal.pone.0264134)
Supplement: S3 Table — (DOCX) [file pone.0264134.s003.docx]

| Question | Responses |
| --- | --- |
| Q1. Is there anything you would like to tell us about the changes that have been brought about by the Covid-19 pandemic and the impact these have had on your mental health or wellbeing? | 702 |
| Q2. What is bothering you the most about the pandemic? What aspects of it have you been finding most difficult? | 2,701 |
| Q3. Has the pandemic had any negative impacts on your mental health and wellbeing? If so could you tell us about these? | 384 |
| Q4. Has the pandemic had any positive impacts on your mental health and wellbeing? If so could you tell us about these? | 69 |
| Q5. Since the Covid-19 pandemic began, how have you been feeling about the future? What are you hopeful or concerned about? | 1,415 |
