# Supplementary figures and images for: Public opinion about the UK government during COVID-19 and implications for public health: A topic modeling analysis of open-ended survey response data
Source: PLoS One. 2022 Apr 14;17(4):e0264134. doi: 10.1371/journal.pone.0264134 (PMC9009625; doi:10.1371/journal.pone.0264134)

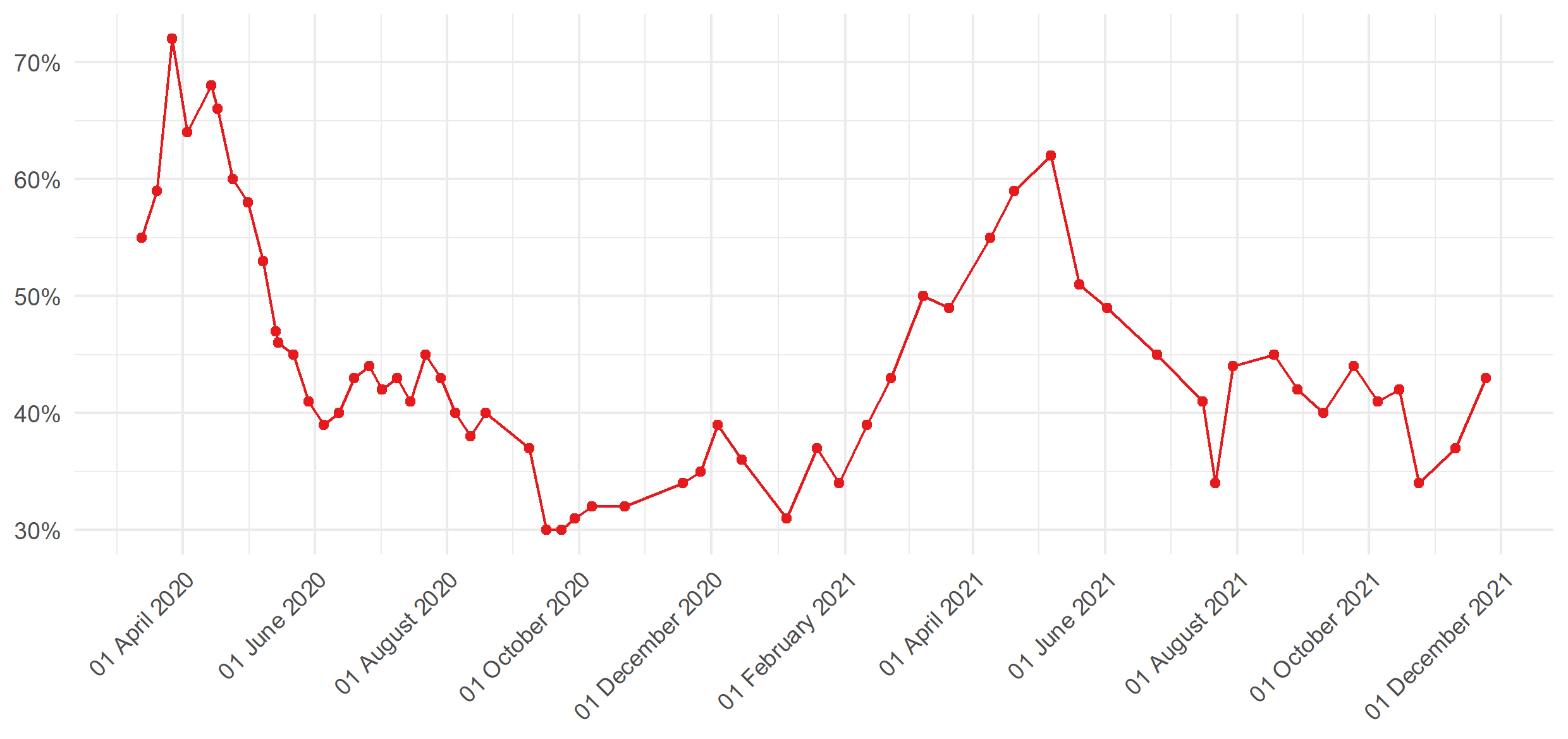

Supplement: S1 Fig — Source: YouGov (2020). (TIFF) [file pone.0264134.s004.tiff]

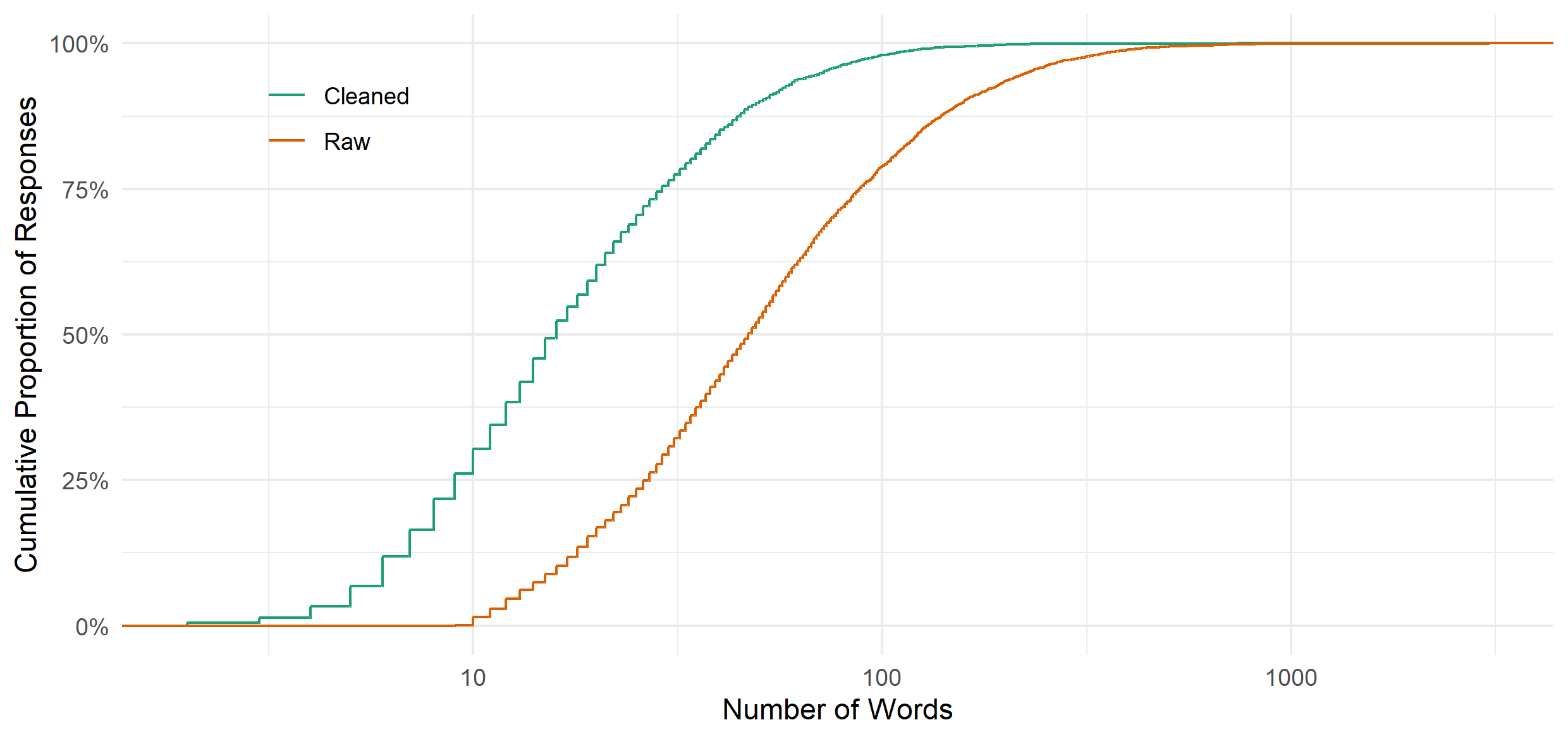

Supplement: S2 Fig — (TIFF) [file pone.0264134.s005.tiff]
