# Supplementary material for: Public opinion about the UK government during COVID-19 and implications for public health: A topic modeling analysis of open-ended survey response data
Source: PLoS One. 2022 Apr 14;17(4):e0264134. doi: 10.1371/journal.pone.0264134 (PMC9009625; doi:10.1371/journal.pone.0264134)
Supplement: S1 File — (PDF) [file pone.0264134.s006.pdf]

Political Context

The period around 14 October to 26 November overlapped with several key developments in the UK Government's response to the COVID-19 pandemic in England. On 12 October, Boris Johnson announced a three-tier system of regional restrictions taking effect from 14 October. One area, Liverpool, was assigned the strictest restrictions. On 22 October, increased financial support for jobs and workers was unveiled, with the package announced shortly after areas of the south of England were placed into higher tiers. On 27 October, the UK recorded 367 deaths from COVID-19, the highest daily total since May. Four days later, the government announced a second four-week national lockdown in England, coming into effect from 5 November. Retail and leisure venues closed and individuals were allowed to meet with at most one member of another household in an outdoor setting. The government's job furlough scheme was extended to the end of March 2021. On 9 November, Pfizer and BioNTech reported effectiveness of 90% in human trials of their COVID-19 vaccine. On 18 November, the National Audit Office released a report finding that politically connected suppliers of PPE were 10 times more likely to be awarded contracts during the pandemic (National Audit Office, 2020), and on 21 November, campaigners announced their intention to take legal action against the UK government over the appointment of politically connected individuals to key roles for tackling COVID-19 (Good Law Project, 2020). On 26 November, a new tier system was announced for England, with news that many parts of the Midlands and the North of England would be placed in the highest tier when the lockdown ended.

References

Good Law Project. (2020). *Jobs for their mates: We're suing*. https://goodlawproject.org/update/jobs-for-mates-jr/

National Audit Office. (2020). *The supply of personal protective equipment (PPE) during the COVID-19 pandemic*. https://www.nao.org.uk/report/supplying-the-nhs-and-adult-social-care-sector-with-personal-protective-equipment-ppe/
