# Supplementary material for: Public opinion about the UK government during COVID-19 and implications for public health: A topic modeling analysis of open-ended survey response data
Source: PLoS One. 2022 Apr 14;17(4):e0264134. doi: 10.1371/journal.pone.0264134 (PMC9009625; doi:10.1371/journal.pone.0264134)
Supplement: S2 File — (PDF) [file pone.0264134.s007.pdf]

Data Cleaning Steps

We performed topic modelling using unigrams (single words). Responses were cleaned using an iterative process. The main steps were as follows. Popular hyphenated words were collapsed into non-hyphenated form and spaces were removed between words that could have been hyphenated (e.g., "pre-pandemic" and "pre pandemic" became "prepandemic"). Punctuation mistakes (e.g., full stops between words) were replaced with whitespace unless the full stop denoted an initialism or a URL. Full stops were removed between initialisms - e.g., U.K. became UK - and "www." was removed from URLs. Spelling mistakes made in 10 or more instances across responses were corrected manually with spelling mistakes were identified using the Hunspell algorithm (Ooms, 2018). All misspellings of "government", "government's" or "governments" were corrected. Accented characters were replaced, contractions expanded (e.g., "hasn't" to "has not"), and possessive forms (trailing "'s") removed. Responses were tokenized into lower-case unigram form and "stop" words (common words such as "the" and "and") were removed. Stop words were identified with the onix (Lextek International, 2021), SMART (Lewis et al., 2004), and snowball (M. Porter & Boulton, 2002) dictionaries. We excluded the stop words "work", "working", "worked", "works", and "help" given their relevance to the current topic. To reduce data sparsity, in the STM analysis, we further stemmed words using the Porter (1980) algorithm, dropped responses if they contained fewer than ten words, and dropped words if they appeared in fewer than five responses (Banks et al., 2018).

Data cleaning was carried out in R version 3.6.3 (R Core Team, 2020) using the tidyverse (Wickham et al., 2019), stringi (Gagolewski, 2020), qdap (Rinker, 2020), hunspell (Ooms, 2018), SnowballC (Bouchet-Valat, 2020), and tidytext (Silge & Robinson, 2016) packages. The code used is available at https://osf.io/jw3gb/. The free-text data are not available due to stipulations set out by the ethics committee.

References

Banks, G. C., Woznyj, H. M., Wesslen, R. S., & Ross, R. L. (2018). A Review of Best Practice Recommendations for Text Analysis in R (and a User-Friendly App). *Journal of Business and Psychology*, *33*(4), 445–459. https://doi.org/10.1007/s10869-017-9528-3

Bouchet-Valat, M. (2020). *SnowballC: Snowball Stemmers Based on the C 'libstemmer' UTF-8 Library* (0.7.0) [Computer software]. https://CRAN.R-project.org/package=SnowballC

Gagolewski, M. (2020). *stringi: Character string processing facilities* (1.5.3) [Computer software]. http://www.gagolewski.com/software/stringi/

Lewis, D. D., Yang, Y., Rose, T. G., & Li, F. (2004). RCV1: A New Benchmark Collection for Text Categorization Research. *The Journal of Machine Learning Research*, *5*, 361–397.

Lextek International. (2021). *Onix Stop Words*. http://www.lextek.com/manuals/onix/stopwords1.html

Ooms, J. (2018). *hunspell: High-Performance Stemmer, Tokenizer, and Spell Checker* (3.0) [Computer software]. https://CRAN.R-project.org/package=hunspell

Porter, M., & Boulton, R. (2002). *Snowball Stop Words*. http://snowball.tartarus.org/algorithms/english/stop.txt

Porter, M. F. (1980). An algorithm for suffix stripping. *Program: Electronic Library and Information Systems*, *14*(3), 130–137. https://doi.org/10.1108/eb046814

R Core Team. (2020). *R: A language and environment for statistical computing* (3.6.3) [Computer software]. R Foundation for Statistical Computing. https://www.R-project.org/

Rinker, T. (2020). *qdap: Quantitative Discourse Analysis Package* (2.4.3) [Computer software]. https://github.com/trinker/qdap

Silge, J., & Robinson, D. (2016). tidytext: Text Mining and Analysis Using Tidy Data Principles in R. *The Journal of Open Source Software*, *1*(3), 37. https://doi.org/10.21105/joss.00037

Wickham, H., Averick, M., Bryan, J., Chang, W., McGowan, L., François, R., Grolemund, G., Hayes, A., Henry, L., Hester, J., Kuhn, M., Pedersen, T., Miller, E., Bache, S., Müller, K., Ooms, J., Robinson, D., Seidel, D., Spinu, V., … Yutani, H. (2019). Welcome to the Tidyverse. *Journal of Open Source Software*, *4*(43), 1686. https://doi.org/10.21105/joss.01686
